# Induction of Autophagy by Vasicinone Protects Neural Cells from Mitochondrial Dysfunction and Attenuates Paraquat-Mediated Parkinson’s Disease Associated α-Synuclein Levels

**DOI:** 10.3390/nu12061707

**Published:** 2020-06-07

**Authors:** Chih-Yang Huang, Kalaiselvi Sivalingam, Marthandam Asokan Shibu, Po-Hsiang Liao, Tsung-Jung Ho, Wei-Wen Kuo, Ray-Jade Chen, Cecilia-Hsuan Day, Vijaya Padma Viswanadha, Da-Tong Ju

**Affiliations:** 1 Department of Medical Research, China Medical University Hospital, China Medical University, Taichung 404, Taiwan; cyhuang@mail.cmu.edu.tw; 2 Department of Biotechnology, Asia University, Taichung 404, Taiwan; 3 Holistic Education Center, Tzu Chi University of Science and Technology, Hualien 970, Taiwan; 4 Cardiovascular and Mitochondria Related Diseases Research Center, Hualien Tzu Chi Hospital, Buddhist Tzu Chi Medical Foundation, Hualien 970, Taiwan; Shibu.m.a@gmail.com; 5 Graduate Institute of Basic Medical Science, China Medical University, Taichung 404, Taiwan; kskalaibiotech@gmail.com; 6 Division of General Surgery, Department of Surgery, Shuang Ho Hospital, Taipei Medical University, New Taipei City 235, Taiwan; robert750927@hotmail.com; 7 Department of Chinese Medicine, Hualien Tzu Chi Hospital, Buddhist Tzu Chi Medical Foundation, Tzu Chi University, Hualien 970, Taiwan; tjho@mail.cmu.edu.tw; 8 Department of Biological Science and Technology, China Medical University, Taichung 404, Taiwan; wwkuo@mail.cmu.edu.tw; 9 Department of Surgery, School of Medicine, College of Medicine, Taipei Medical University, Taipei 110, Taiwan; rayjchen@tmu.edu.tw; 10 Department of Nursing, MeiHo University, Pingtung 91202, Taiwan; x00003023@mail.meiho.edu.tw; 11 Department of Biotechnology, Bharathiar University, Tamilnadu 641046, India; padma.vijaya@gmail.com; 12 Department of Neurological Surgery, Tri-Service General Hospital, National Defense Medical Center, Taipei 114, Taiwan

**Keywords:** Parkinson’s disease, vasicinone, paraquat, α-synuclein, mitophagy, reactive oxygen species

## Abstract

Mitochondrial dysfunction and disturbed mitochondrial dynamics were found to be common phenomena in the pathogenesis of Parkinson’s disease (PD). Vasicinone is a quinazoline alkaloid from *Adhatoda vasica*. Here, we investigated the autophagy/mitophagy-enhancing effect of vasicinone and explored its neuroprotective mechanism in paraquat-mimic PD modal in SH-SY5Y cells. Vasicinone rescued the paraquat-induced loss of cell viability and mitochondrial membrane potential. Subsequently, the accumulation of mitochondrial reactive oxygen species (ROS) was balanced by an increase in the expression of antioxidant enzymes. Furthermore, vasicinone restored paraquat-impaired autophagy and mitophagy regulators DJ-1, PINK-1 and Parkin in SH-SY5Y cells. The vasicinone mediated autophagy pathways were abrogated by treatment with the autophagy inhibitor 3-MA, which lead to increases α-synuclein accumulation and decreased the expression of p-ULK and ATG proteins and the autophagy marker LC3-II compared to that observed without 3-MA treatment. These results demonstrated that vasicinone exerted neuroprotective effects by upregulating autophagy and PINK-1/Parkin mediated mitophagy in SH-SY5Y cells.

## 1. Introduction

Parkinson’s disease (PD) is the second most common neurodegenerative disorder, affecting approximately 3.4% of the world’s population over 60 years of age [1,2]. It is characterized by the progressive loss of dopaminergic neurons in the substantia nigra pars compacta of the midbrain [3] and the aggregation of α-synuclein, which is a defining pathological characteristic of the disease [4]. Motor defects (static tremor, postural imbalance, bradykinesia, and muscle rigidity) and nonmotor defects (progressive impairment of cognitive function, sleep disturbances and depression) are commonly reported symptoms in PD patients [5]. The central objective in treating patients with PD is to increase the striatal dopamine content and prevent further degeneration of dopamine-producing neurons in the substantia nigra region of the ventral midbrain. Current therapies for PD are the administration of a dopamine precursor, anticholinergic agents, dopamine agonists, and levodopa (L-DOPA), which provide symptomatic relief for a few years [5,6]. However, their long-term usage is associated with a progressive decrease in drug response, motor fluctuations, dyskinesias, drug-associated toxicity and failure to prevent the progression of the disease [6]. The intricate pathology of PD and the lack of long-term treatment options continue to be major restrictions in the treatment of PD. This scenario has inducing researchers to investigate novel targets and treatment strategies. 

The aggregation of α-synuclein and α-synuclein-containing inclusion Lewy bodies is the major hallmark of PD [7,8]. Several studies have reported that aggregates of abnormal proteins or injured organelles can be removed through the autophagy machinery [9,10]. Consequently, the impairment of autophagy degradation systems may be an important therapeutic strategy in PD. Autophagosomal formation involves multiple autophagy-related (ATG) proteins and the conversion of LC3-I to LC3-II. The autophagosome subsequently fuses with a lysosome to generate an autolysosome, and the cytosolic proteins and damaged organelles are degraded by lysosomal enzymes in the autolysosome [11].

Previous reports suggested that defects in mitochondrial dynamics such as fission, fusion, transport, biogenesis, and degradation through mitophagy may be involved in the pathogenesis of PD progression [12,13,14,15]. Excessive misfolded proteins and/or mitochondrial dysfunction deplete ATP and have been implicated in the pathogenesis of PD [16]. Mitophagy selectively removes dysfunctional or damaged mitochondria and maintains a healthy mitochondria population [17]. Phosphatase and tensin homolog-induced putative kinase 1 (PINK-1) and the Parkin-mediated mitophagy pathway are involved in eliminating depolarized mitochondria [18,19]. In healthy mitochondria, PINK-1 is imported via its mitochondrial targeting sequence from the cytosol to the mitochondria, where it is cleaved by mitochondrial matrix proteases. Upon the loss of the mitochondrial membrane potential (Δψm), PINK1 is stabilized instead at the mitochondrial outer membrane to recruit Parkin into target mitochondria, which promotes the removal of damaged mitochondria via mitophagy [20]. Insufficient mitophagy induces the accumulation of dysfunctional mitochondria, contributing to the pathogenesis of diseases characterized by dysfunctional energy metabolism [21,22]. Mutations in PINK-1 and Parkin are associated with mitochondrial dysfunction and neurodegenerative diseases [22]. The enhanced clearance of misfolded proteins and defective mitochondria by mitophagy plays a important role in neuroprotection [23].

*Adhatoda vasica Nees* belongs to the medicinal family *Acanthaceae* and is commonly known as vasaka. Previous studies have reported that extracts of *A. vasica* exert many beneficial effects by its bronchodilator [24], anti-inflammatory [25], anticholinesterase [26] and anticancer activities [27,28]. Vasicinone is a biologically active quinazoline alkaloid isolated from leaf extracts of *A. vasica*.

Previous studies reported that neurotoxins such as rotenone, 1-methyl-4-phenyl-1,2,3,6- tetrahydropyridine (MPTP), and 6-hydroxydopamine (6-OHDA) have been implicated in the dysregulation of autophagy in SH-SY5Y cells and in mice [29,30,31,32]. In this study, we exposed SH-SY5Y cells to paraquat to mimic a cellular model of PD. The protective effect of vasicinone and its possible molecular mechanism were investigated in this model. We found that vasicinone significantly protect the SH-SY5Y cells from paraquat mediated toxicity, enhancing the clearance of misfolded proteins and defective mitochondria via autophagy.

## 2. Material and Methods

### 2.1. Chemicals

Vasicinone (purity > 98%) was purchased from Cayman Chemical (CAS-486-64-6, Ann Arbor, MI, USA). Paraquat, a Mitochondria Staining Kit (JC-1 stain) and 3-(4,5-dimethyl-2-thiazolyl)-2,5-diphenyltetrazolium bromide (MTT) were purchased from Sigma-Aldrich (St. Louis, MO, USA). The MitoSOXTM Red kit (M36008) was purchased from Thermo Fisher Scientific (Waltham, MA, USA). Dulbecco’s modified Eagle medium (DMEM:F12) and fetal bovine serum (FBS) were purchased from Gibco (Grand Island, NY, USA). Primary antibodies against SOD-1, SOD-2, GST, GPx, TOM-20, VDAC-1, Parkin, PINK-1 and GAPDH were purchased from Santa Cruz Technology (Dallas, TX, USA), antibodies against DJ-1, α-synuclein, p-ULK, ATG7, ATG12 and LC3B were purchased from Cell Signaling Technology (Danvers, MA, USA) and an antibody against Nrf-2 was purchased from Abcam (Cambridge, MA, USA). All fluorescent secondary antibodies were purchased from Thermo Fisher Scientific in the USA. 

### 2.2. Cell Culture and Treatments

SH-SY5Y cells were obtained from the American Type Culture Collection (ATCC) and maintained in a Dulbecco’s modified Eagle medium/F12 nutrient mixture (DMEM:F12) with L-glutamine (Gibco, Gaithersburg, MD, USA) supplemented with heat-inactivated fetal bovine serum (FBS) (10% v/v) and penicillin–streptomycin (1% v/v) at 37 °C under a humidified atmospheric condition containing 5% CO_2_. Vasicinone was prepared in dimethylsulfoxide (DMSO), and paraquat was dissolved in phosphate buffered saline (PBS) and then stored at −20 °C. Vasicinone and paraquat were further diluted in PBS to obtain working concentrations. To investigate the effect of vasicinone on paraquat-induced neurotoxicity, SH-SY5Y cells were pretreated with vasicinone for 24 h followed by incubation with paraquat for another 24 h.

### 2.3. MTT Assay

The viability of SH-SY5Y cells was measured by MTT assay. Briefly, the cells were seeded into 96-well plates at a density of 1 × 10^4^ cells/well in 200 μL of medium. When the cells reached 70–80% confluence, they were pretreated with vasicinone for 24 h followed by incubation with paraquat for another 24 h. After treatment for 48 h, the cells were incubated with 20 µL of an MTT (5 mg/mL in PBS) solution for another 4 h at 37 °C. Then, formazan crystals were dissolved in DMSO, and the absorbance at 570 nm was measured using a microplate ELISA reader (Bio-Tek Instruments, Winooski, VT, USA). All experiments were performed independently in triplicate (Tokyo, Japan).

### 2.4. Detection of Mitochondrial ROS Generation

Mitochondrial ROS generation were measured by using MitoSOX Red (M36008, Invitrogen, Carlsbad, CA, USA). MitoSOX Red is a live cell permeant that selectively targets mitochondria and exhibits red fluorescence (with excitation at 510 nm and emission at 580 nm) after it is oxidized by superoxide. Briefly, the drug treated cells were washed with PBS and incubated with 2.5 µM MitoSOX Red for 30 min at 37 °C and 5% CO_2_. Then, the cells were washed with PBS, and red fluorescence was measured using a fluorescence microscope (Zeiss, Chicago, IL, USA).

### 2.5. Detection of Mitochondrial Membrane Potential (Δψm)

A JC-1 kit (CS0390, Sigma, St. Louis, MO, USA) was used to measure mitochondrial depolarization in SH-SY5Y cells according to the manufacturer’s instructions. A cell-permeable cationic dye, 5,5′,6,6′-tetrachloro-1,1′,3,3′-tetraethylbenzimidazolocarbocyanine iodide (JC-1), can enter and accumulate within mitochondria. JC-1 aggregation shows red fluorescence in healthy cells with a normal membrane potential (Δψm). In apoptotic cells, the loss of Δψm causes the cationic dye to remain in the cytoplasm in its monomeric form and exhibit green fluorescence. Briefly, cells were seeded into 12-well plates at a density of 1 × 10^5^ cells/well. After the treatment period, cells were washed with PBS and incubated with 500 μL of JC-1 staining solution for 20 min at 37 °C in 5% CO_2_. After washing with PBS, the green (excitation, 490 nm; emission, 530 nm) and red (excitation, 525 nm; emission, 590 nm) fluorescence intensities were measured with an inverted fluorescence microscope.

### 2.6. Seperation of Mitochondrial Fractionation

Mitochondria were isolated from SH-SY5Y cells with a kit according to the manufacturer’s instructions (Mitochondria Isolation Kit, Thermo Fisher Scientific, Waltham, MA, USA). Briefly, at the end of the treatment period, the collected cells were suspended in mitochondria isolation reagent A and incubated for 2 min on ice. After incubation, 10 μL of reagent B was added to the cells, which were incubated for 5 min on ice. After incubation, 800 μL of reagent C was added to the tube, followed by centrifugation at 700× *g* for 10 min at 4 °C. Then, the supernatant from each sample was collected in a new tube and centrifuged at 3000× *g* for 15 min at 4 °C. After centrifugation, the pellets (mitochondria fraction) were retained to do further mitochondrial protein expression analysis.

### 2.7. Immunofluorescence Staining

Briefly, cells were fixed with 4% paraformaldehyde for 15–30 min at room temperature followed by permeabilization with 0.2% Triton X-100 in PBS for 15 min at room temperature. Then, the cells were incubated with 5% normal goat serum for 1 h to block nonspecific binding. The appropriate primary antibody (diluted 1: 300) was subsequently added, and the cells were incubated overnight at 4 °C. After washing with PBS, the cells were incubated with specific secondary antibodies (Invitrogen). The cellular nuclei were stained with 4′6-diamidino-2-phenylindole (DAPI). Slides were examined with a fluorescent inverted phase-contrast microscope (LSM 510; Zeiss, Chicago, IL, USA).

### 2.8. Immunoblotting Assay

The collected cell lysates were centrifuged at 12,000× *g* for 30 min at 4 °C. The protein concentrations were estimated by the Bradford method (Bio-Rad, Hercules, CA, USA). Equivalent amounts of proteins from each group were separated with 8–12% SDS-polyacrylamide gel electrophoresis and transferred to a polyvinylidene difluoride membrane (GE, Amersham, UK). After protein transfer to the membrane, the membrane was blocked with 5% nonfat milk in Tris buffered saline (TBST) at room temperature for 1 h and incubated overnight with specific primary antibodies at 4 °C. After washing with TBST, the blots were incubated with the appropriate horseradish peroxidase-conjugated secondary antibody for 1 h, and then bands were visualized using enhanced chemiluminescent Horseradish Peroxidase (HRP) substrate (Millipore, Billerica, MA, USA). To ensure equal protein loading, GAPDH was used as an internal control. Densitometric analysis was carried out using ImageJ software (NIH, Bethesda, MD, USA).

### 2.9. Statistical Analysis

Statistical analyses were performed using SPSS software 17.0 (SPSS Inc., Chicago, IL, USA). All results are expressed as the mean ± standard deviation (SD). Data were analyzed by one-way analysis of variance (ANOVA), followed by Tukey’s post hoc test. *p* < 0.05 indicated statistical significance. All results were quantified using ImageJ software (NIH, Bethesda, MD, USA).

## 3. Results

### 3.1. Vasicinone Ameliorated Paraquat Mediated Cytotoxicity in SH-SY5Y Cells

**Figure 1:**
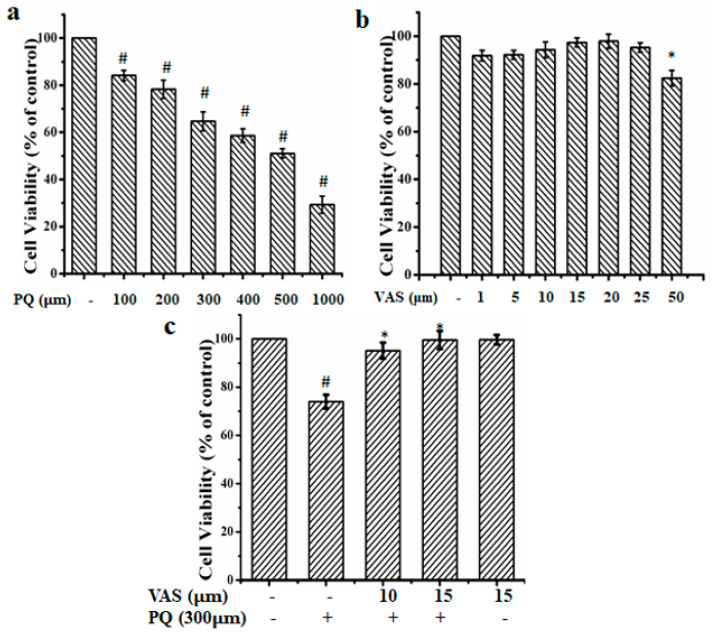
Effects of paraquat (PQ) and vasicinone (VAS) on SH-SY5Y cell viability. (**a**) Cells were incubated with different concentrations of paraquat ranging from 0.1 mM to 1 mM for 24 h for the 3-(4,5-dimethylthiazol-2-yl)-2,5-diphenyl tetrazolium bromide (MTT) assay. The data are expressed as the mean ± standard deviation (SD), *n* = 3. ^#^ *p* < 0.01, significantly different from control cells. (**b**) Cells were treated with 1 to 50 μΜ of vasicinone for 24 h. Cell viability was assessed and expressed as a % compared to the viability of the control group. The data are expressed as the mean ± SD, *n* = 3. * *p* < 0.01, significantly different from control cells. (**c**) Cells were pretreated with 10 and 15 μΜ vasicinone for 24 h followed by incubation with 300 μM paraquat for another 24 h. Cell viability was assessed and expressed as a % compared to the viability of the control group. The data are expressed as the mean ± SD, *n* = 3. ^#^ *p* < 0.01, significantly different from control cells; * *p* < 0.01, compared with paraquat-treated group by one-way ANOVA.

The viability of SH-SY5Y cells were detected using the conventional 3-(4,5-dimethylthiazol-2-yl)-2,5-diphenyl tetrazolium bromide (MTT) assay. As shown in Figure 1a, paraquat treatment significantly reduced the viability of SH-SY5Y cells in a dose-dependent manner (100–1000 µM) compared to that observed in untreated cells. Based on the MTT results, we chooses 300 µM of paraquat concentration to mimic PD model. As shown in Figure 1b, the treatment of vasicinone (1, 5, 10, 15, 20 and 25 µM) had no significant cytotoxic effect on cell viability upto 25 µM. Further to finalize the effect doses of vasicinone, cells were pretreated with different concentrations of vasicinone for 24 h, followed by incubation with paraquat (300 µM) for another 24 h. We found that treatment with 10 µM and 15 µM vasicinone significantly attenuated the paraquat-induced loss of cell viability (Figure 1c).

### 3.2. Vasicinone Prevented Paraquat Mediated Loss of Mitochondriyal Potential and ROS Generation in SH-SY5Y Cells

**Figure 2:**
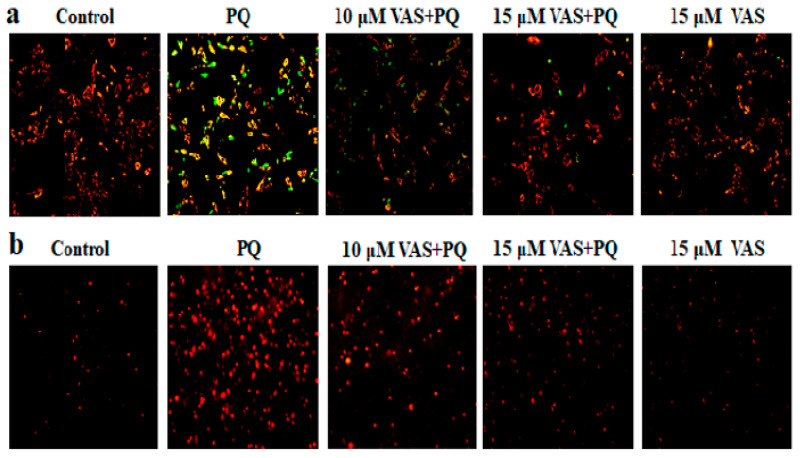
Effect of vasicinone on paraquat-mediated mitochondrial dysfunction in SH-SY5Y cells. Cells were pretreated with vasicinone for 24 h followed by incubation with 300 μM paraquat for another 24 h. (**a**) The mitochondrial membrane potential was assessed by the labeling of mitochondria with JC-1 dye. (**b**) Mitochondrial reactive oxygen species (ROS) levels were determined using the fluorescent dye MitoSOX Red, a mitochondrial superoxide indicator.

To measure the mitochondrial membrane potential, SH-SY5Y cells were incubated with JC-1 stain to specifically tag the mitochondria. JC-1 aggregates in healthy mitochondria and fluoresces red, whereas in depolarized mitochondria with a low membrane potential, it fluoresces green. We found that paraquat-treated cells exhibited increased green fluorescence compared to untreated cells that was significantly inhibited by pretreatment with 10 μM and 15 μM vasicinone and emitted a strong, red fluorescence signal (Figure 2a).

We subsequently examined paraquat-mediated mitochondrial ROS generation by using the fluorescent dye MitoSOX (C43H34N3IP), which is membrane-permeable and rapidly targets mitochondrial superoxide. The excessive production of mitochondrial ROS disrupts normal redox signaling. As shown in Figure 2b, treatment with vasicinone dramatically decreased the mitochondrial ROS generation induced by paraquat. Treatment with vasicinone alone did not cause significant ROS generation. However, vasicinone treatment significantly attenuates the paraquat-mediated loss of mitochondrial membrane potential and ROS generation, demonstrating that vasicinone might be restores the mitochondrial function in SH-SY5Y cells.

### 3.3. Vasicinone Protected SH-SY5Y Cells through Maintaining the Antioxidant Redox System

**Figure 3:**
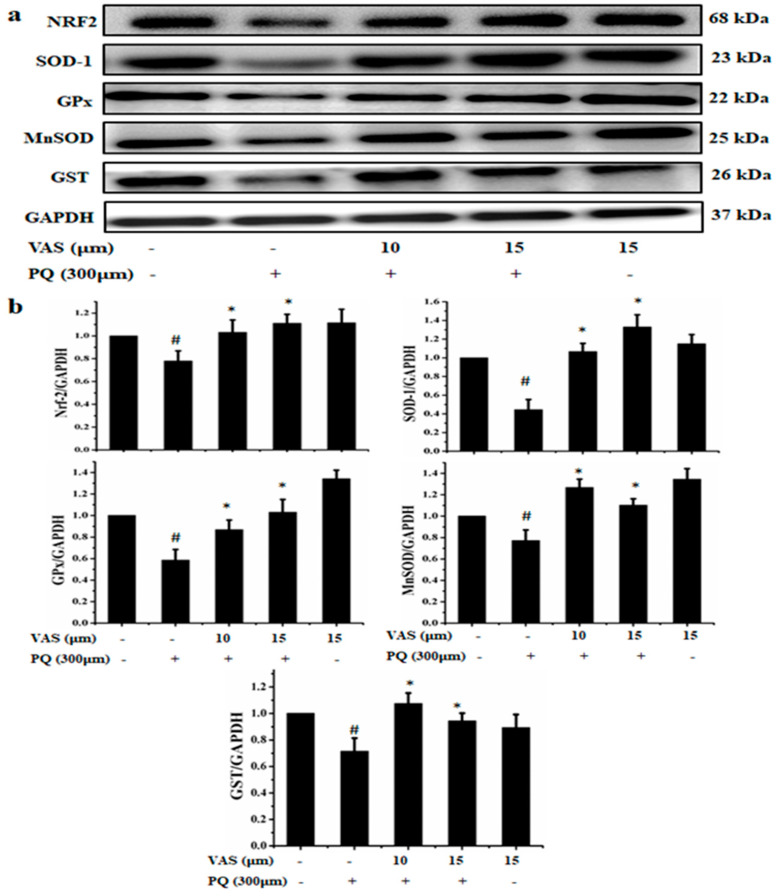
Effect of vasicinone (VAS) on the expression of antioxidant regulators in SH-SY5Y cells. (**a**) Cells were pretreated with vasicinone for 24 h, and paraquat (PQ) (300 μM) was added for an additional 24 h. The expression levels of the antioxidant proteins Nrf2, SOD-1, GPx, MnSOD and GST were measured using Western blotting. GAPDH (glyceraldehyde 3-phosphate dehydrogenase) was used as an internal standard protein. Three independent experiments were performed. (**b**) Nrf2, SOD-1, GPx, MnSOD and GST were determined by densitometry of the bands intensity. Three independent experiments were performed for this assay. Results are shown as the mean ± SD. ^#^ *p* < 0.05, compared with control cells; * *p* < 0.05, compared with paraquat-treated cells.

Vasicinone increases the expression of antioxidant proteins to maintain a balanced redox state to prevent oxidative stress mediated by paraquat in SH-SY5Y cells. As shown in Figure 3, paraquat treatment significantly reduced the expression of the antioxidant proteins Nrf2, SOD-1, GPx, MnSOD and GST compared to that in the control. Pretreatment with 10 µM and 15 µM vasicinone significantly restored the antioxidant status in a dose-dependent manner (Figure 3a,b).

### 3.4. Vasicinone Enhanced the Clearance of α-Synuclein by Upregulating Autophagy

**Figure 4:**
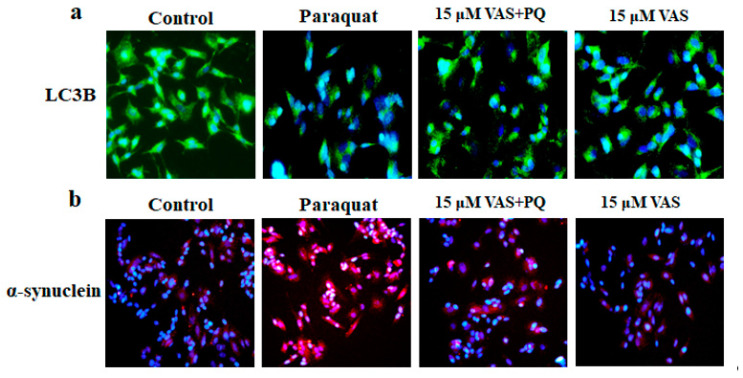
Vasicinone (VAS) enhanced the clearance of α-synuclein by upregulating the autophagy marker LC3. SH-SY5Y cells were pretreated with vasicinone for 24 h, followed by incubation with 300 μM paraquat (PQ)for another 24 h. (**a**) Immunostaining analysis of three independent experiments showed the expression of LC3 (green) in different groups. (**b**) SH-SY5Y cells were immunostained for α-synuclein (red). DAPI staining was used to indicate the position of the nuclei (blue). Scale bars = 50 μm.

Immunofluorescence staining showed that paraquat notably decreased the expression of LC3 (fluorescent green) and increased α-synuclein (fluorescent red) accumulation in SH-SY5Y cells compared to that observed in untreated cells. At the same time, the pretreatment of SH-SY5Y cells with vasicinone followed by incubation with paraquat decreased the α-synuclein accumulation and increased LC3 expression shows puncta-like appearances compared to paraquat alone treated cells (Figure 4a,b). 

**Figure 5:**
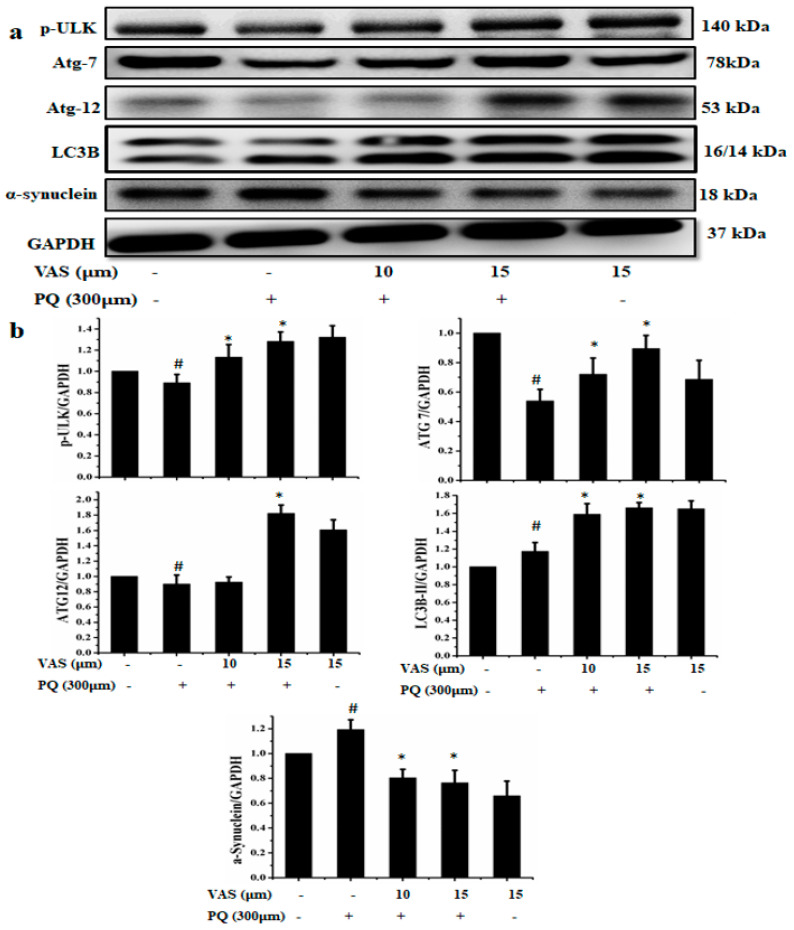
Effect of vasicinone (VAS) against paraquat (PQ) mediated neurotoxicity in SH-SY5Y cells via the induction of autophagy. SH-SY5Y cells were pretreated with vasicinone for 24 h, followed by incubation with 300 μM paraquat for another 24 h. (**a**) The protein expression of the autophagy-associated proteins p-ULK, ATG7, ATG12, LC3B and α-synuclein was determined by Western blotting. GAPDH was used as the loading control. (**b**) p-ULK, ATG7, ATG12, LC3B and α-synuclein were determined by densitometry of the of the blots. Three independent experiments were performed for this assay. Results are shown as the mean ± SD. ^#^ *p* < 0.05, compared with control cells; * *p* < 0.05, compared with paraquat-treated cells.

The immunoblotting results showed that vasicinone significantly enhanced the protein expression of p-ULK, ATG7, and ATG12 and increased LC3B, indicating that vasicinone had the potential effects to induce autophagy. The formation of autophagosomes and autophagolysosomes, referred to as autophagic vacuoles (AVs), is considered the characteristic component of autophagy. LC3 is required for the formation of autophagosomal membranes, and the conversion of LC3B-I to LC3B-II occurs during autophagy induction, with the latter considered an autophagosomal marker in mammalian cells. Previous studies reported that the disruption of the autophagy pathway lead to Parkinson’s disease and α-synuclein accumulation. Paraquat treatment significantly increased the accumulation of α-synuclein compared to that observed in the control, and pretreatment with 10 µM and 15 µM vasicinone significantly reduced α-synuclein expression (Figure 5a,b).

**Figure 6:**
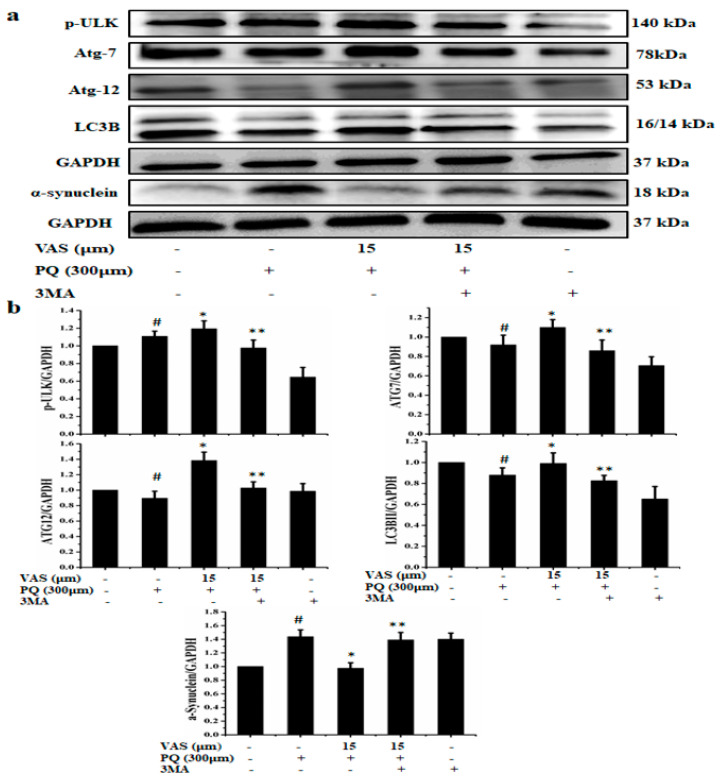
Inhibition of autophagy by treatment with 3MA (3-Methyladenine) (10 mM) 1 h prior to vasicinone (VAS) treatment. (**a**) The protein expression levels of p-ULK, ATG7, ATG12, LC3B and α-synuclein were analyzed by Western blotting. GAPDH was used as an internal standard protein. (**b**) Expression of p-ULK, ATG7, ATG12, LC3B and α-synuclein were analyzed by densitometry of bands. Three independent experiments were performed. Results are shown as the mean ± SD. ^#^ *p* < 0.05, compared with control cells; * *p* < 0.05, compared with paraquat-treated cells; ** *p* < 0.05, compared with (vasicinone + paraquat (PQ)) treated group.

To confirm the vasicinone-activated autophagy pathway, cells were treated with autophagy inhibitor 3-MA. The autophagy-associated proteins p-ULK, ATG7, ATG12, LC3B, and the α-synuclein expression were analyzed by Western blotting. The protein expression levels of p-ULK, ATG7, ATG12 and LC3B were decreased in SH-SY5Y cells pretreated with 3-MA compared to those observed in untreated cells. The addition of 3-MA abolished the ameliorating effect of vasicinone by decreasing the expression of autophagy inducing proteins and increases the expression of α-synuclein in the vasicinone-, paraquat-, and 3-MA-treated cells compared with the vasicinone- and paraquat-treated cells (Figure 6a,b).

### 3.5. Vasicinone Protected SH-SY5Y Cells through PINK-1/Parkin-Mediated Mitophagy

**Figure 7:**
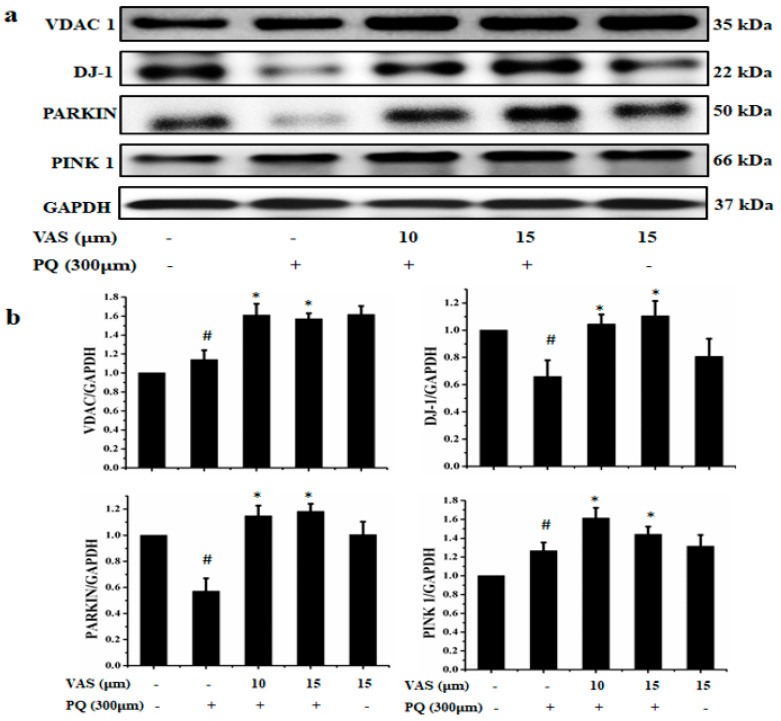
Protective effect of vasicinone (VAS) by the induction of mitophagy in SH-SY5Y cells. (**a**) Cells were pretreated with vasicinone for 24 h, followed by exposure to 300 μM paraquat (PQ) for another 24 h. Mitochondrial fractions were prepared to determine the expression of the mitochondrial proteins VDAC-1, DJ-1, Parkin and PINK-1. GAPDH was used as an internal control protein. (**b**) Expression levels determined by densitometry. The experiment was performed in triplicate. ^#^ *p* < 0.05, compared with control cells; * *p* < 0.05, compared with paraquat-treated cells.

Mitochondria are the essential source of ATP and reactive oxygen species (ROS) in mammalian cells. We found that treatment with paraquat disrupted the mitochondrial proteins, which may lead to the pathogenesis of PD. We examined the effect of vasicinone on the mitophagy regulators DJ-1, PINK-1, Parkin and VDAC-1 in SH-SY5Y cells. Treatment with paraquat disturbed the expression of the mitophagy regulators DJ-1, PINK-1, Parkin and VDAC-1 compared to that in untreated cells. Vasicinone treatment subsequently restored paraquat-mediated impaired mitophagy to protect the cells (Figure 7a,b).

## 4. Discussion

Recent research has focused on identifying potent neuroprotective candidate agents for the disease-modifying therapy of several neurodegenerative diseases. The present study reported, for the first time, the neuroprotective potential of vasicinone against paraquat-mediated PD in SH-SY5Y cells. Our findings explored the neuroprotective effect of vasicinone, which ameliorated paraquat-mediated SH-SY5Y cell damage by upregulating autophagy, increasing cell viability, decreasing paraquat-mediated mitochondrial ROS generation and inhibiting the loss of the mitochondrial membrane potential. Furthermore, vasicinone attenuated the accumulation of α-synuclein and restored intracellular antioxidant levels in SH-SY5Y cells. However, the effect of vasicinone in differentiated SH-SY5Y cells will helps to prove the efficacy of vasicinone in neuron-like cells, so far, which is limitation of this current study.

Autophagy is an essential self-degradative process in which misfolded proteins and damaged organelles are delivered to the lysosome for degradation. Autophagic pathways include macroautophagy, chaperone-mediated autophagy and microautophagy, and each pathway involves different mechanisms by which the substrate is delivered to the lysosome for degradation. The impairment of these pathways and the resulting accumulation of protein aggregates represent a common pathobiological feature of neurodegenerative disorders [33]. Neurons require a basal level of autophagic degradation to mediate the replacement of damaged organelles and to facilitate synaptic remodeling. Under disease conditions, neuronal loss in the substantia nigra is partly due to the accumulation of aggregated and/or misfolded proteins [34]. Most aggregated and/or misfolded proteins are degraded by the ubiquitin proteasome system and the autophagy–lysosome pathway [35]. Previous studies stated that resveratrol activates autophagy, which helps to degrade the α-synuclein aggregates in PC12 cells [36]. González-Polo et al. reported that paraquat induces the accumulation of double-membrane autophagic vacuoles (AVs) in the cytoplasm of SH-SY5Y cells [37]. In the same line, vasicinone also activates autophagy and promotes the degradation of accumulated proteins induced by paraquat in SH-SY5Y cells. The inhibition of autophagy by 3-MA significantly decreases the autophagy and abolished the vasicinone-afforded protection against paraquat mediated neurotoxicity.

Mitochondrial dysfunction is considered a critical mechanism underlying the pathogenesis of PD [38,39]. Markedly, excess ROS generation induces the oxidative stress also enhances mitochondrial dysfunction, which leads to neuronal damage [40]. Several studies reported that mitochondria are major source of paraquat-induced oxidative stress in neuronal cells [41,42]. Mitophagy is essential for the control of mitochondrial quality, as mitophagy impairment results in the persistence of damaged mitochondria and intracellular ROS accumulation. Mitochondrial autophagy selectively removes damaged and dysfunctional mitochondria, which plays an important role in maintaining mitochondrial homeostasis and preventing cell death [43,44]. The PINK-1-dependent activation of the Parkin pathway is involved in eliminating depolarized mitochondria [45]. Mutation of PINK-1 or Parkin causes early onset PD [46]. Previous studies also reported that neurotoxins, including MPTP, 6-OHDA, dopamine and rotenone affects mitochondrial parkin. The mammalian voltage-dependent anion channels (VDACs) serve as mitochondrial docking sites to interact with Parkin on defective mitochondria, which will help to subsequent mitophagy [47]. Here, we demonstrated that vasicinone pretreatment reduced paraquat-mediated mitochondrial dysfunction via activation of PINK-1/parkin.

DJ-1 is a redox sensor for oxidative stress and will helps to maintain mitochondrial complex I activity and integrity. The loss of DJ-1 expression impairs the oxidative phosphorylation, mitochondrial membrane potential and degradation of misfolded proteins leads to mitochondrial dysfunction associated PD pathogenesis [48,49]. Further, DJ-1 is a novel antioxidant regulator that protects cells from oxidative stress and induces Nrf2 expression [50]. Nrf-2 regulates the antioxidant enzymes, metabolic pathways, mitochondrial bioenergetics and autophagy [51]. We found that vasicinone significantly restored the paraquat-induced changes in the protein expression of DJ-1 and Nrf-2 in SH-SY5Y cells.

There is long-standing evidence that a chronic imbalance between free radicals and antioxidant defense contributes to many pathological processes and disease conditions, such as PD [52]. In addition, free radicals are neutralized by an elaborate antioxidant defense system consisting of enzymes such as SOD and GPx and nonenzymatic antioxidants such as GSH [53]. Vasicinone improved free radical scavenging and antioxidant activity in the PD cell model mimicked by paraquat.

Moreover, Biosa et al. reported that PINK1 and Parkin-deficient cells increase mitochondrial fragmentation compare to wild-type cells SH-SY5Y cells. Remarkably, either SOD1 or SOD2 overexpression restores the redox state of mitochondria and partially rescue the mitochondria fragmentation [54]. Interestingly our recent studies also showed that paraquat was counteracted by vasicinone treatment, which activates the IGF-1R/AKT/PI3K signaling pathway to inhibit MAP kinases and apoptotic cell death [55]. The human dopaminergic cell line SH-SY5Y has been widely used in vitro model for PD and neurotoxicity experiments.

In summary, our findings demonstrate that vasicinone induces autophagy to ameliorate paraquat mediated SH-SY5Y cell damage, inhibit intracellular α-synuclein accumulation, increase antioxidants of Nrf-2, SOD, GPx and GST, prevent mitochondrial ROS generation and ameliorate loss of Δψm. We also found that vasicinone activates PINK1-Parkin dependent mitochondrial autophagy/mitophagy to maintain mitochondrial homeostasis and preventing cell death. Based on our findings, vasicinone is a potential candidate for further in vivo studies to explore insight of mechanistic aspects aimed at the treatment of PD. In the present study, we demonstrated that paraquat caused mitochondrial dysfunction and impaired autophagic activity. However, vasicinone treatment induces autophagic activity and rescued mitochondrial dysfunction.
